# Liver necrosis shortly after pancreaticoduodenectomy with resection of the replaced left hepatic artery

**DOI:** 10.1186/s12957-017-1151-2

**Published:** 2017-04-11

**Authors:** Michihiro Yamamoto, Masazumi Zaima, Hidekazu Yamamoto, Hideki Harada, Junichiro Kawamura, Masahiro Yamada, Tekefumi Yazawa, Junya Kawasoe

**Affiliations:** Shiga Medical Center for Adults, 4-30 Moriyama 5-chome, Moriyama city, Shiga Prefecture 524-8524 Japan

**Keywords:** Massive hepatic necrosis, Pancreaticoduodenectomy, Hepatic artery, Ischemia

## Abstract

**Background:**

Surgeons, in general, underestimate the replaced left hepatic artery (rLHA) that arises from the left gastric artery (LGA), compared with the replaced right hepatic artery (rRHA), especially in standard gastric cancer surgery. During pancreaticoduodenectomy (PD), preservation of the rRHA arising from the superior mesenteric artery (SMA) is widely accepted to prevent critical postoperative complications, such as liver necrosis, bile duct ischemia, and biliary anastomotic leakage. In contrast, details of complication onset following rLHA resection remain unknown. We report two cases of postoperative liver necrosis shortly after rLHA resection during PD for advanced gastric cancer.

**Case presentation:**

Both cases had advanced gastric cancer with infiltration of the pancreatic head. In case 1, the rLHA comprised segment 2/3 artery (A2 + A3), which arose from the LGA. The rRHA originated from the SMA, and the segment 4 artery (A4) was a branch of the rRHA. We conducted PD with combined en bloc resection of both the rLHA and rRHA, and anastomosis between the distal and proximal stumps of the rRHA and LGA, respectively. The divided A2 + A3 was not reconstructed. In case 2, the rLHA comprised segment 2 artery (A2) only, which arose from the LGA. The segment 3/4 artery and the RHAs originated from the proper hepatic artery. We undertook PD with combined en bloc resection of A2 without vascular reconstruction. In both patients, serious necrosis of the lateral segment of the liver occurred within 6 days after PD. Case 1 recovered with conservative management, whereas case 2 required lateral segmentectomy of the liver. Pathologically, the necrotic area in case 2 was apparently circumscribed and confined to segment 2 of the liver, potentially implicating rLHA resection during PD as causing hepatic necrosis.

**Conclusions:**

During PD, rLHA resection can cause serious liver necrosis. Therefore, this artery should be preserved as far as oncologically acceptable. In cases that require ﻿rLHA resection during PD due to tumor conditions, surgeons should carefully monitor postoperative course while keeping in mind the possible necessity of urgent hepatectomy.

## Background

During standard gastric cancer surgery, the replaced left hepatic artery (rLHA) arising from the left gastric artery (LGA) is generally underestimated, compared with the replaced right hepatic artery (rRHA). The rLHA is often resected for en bloc lymphadenectomy without vascular reconstruction in gastric cancer surgery despite a reported risk of ischemic liver injury [1–4].

During pancreaticoduodenectomy (PD), preservation of the rRHA arising from the superior mesenteric artery (SMA) is widely accepted [5–10]. Intraoperative damage to the rRHA can cause bile duct or liver ischemia, biliary anastomotic leakage, liver necrosis, and mortality [11]. In contrast, such complications that potentially cause postoperative liver necrosis have not been reported after resection of the rLHA arising from the LGA during PD. We encountered two cases of postoperative liver necrosis due to rLHA resection during PD for advanced gastric cancer.

## Case presentation

### Case 1

**Fig. 1:**
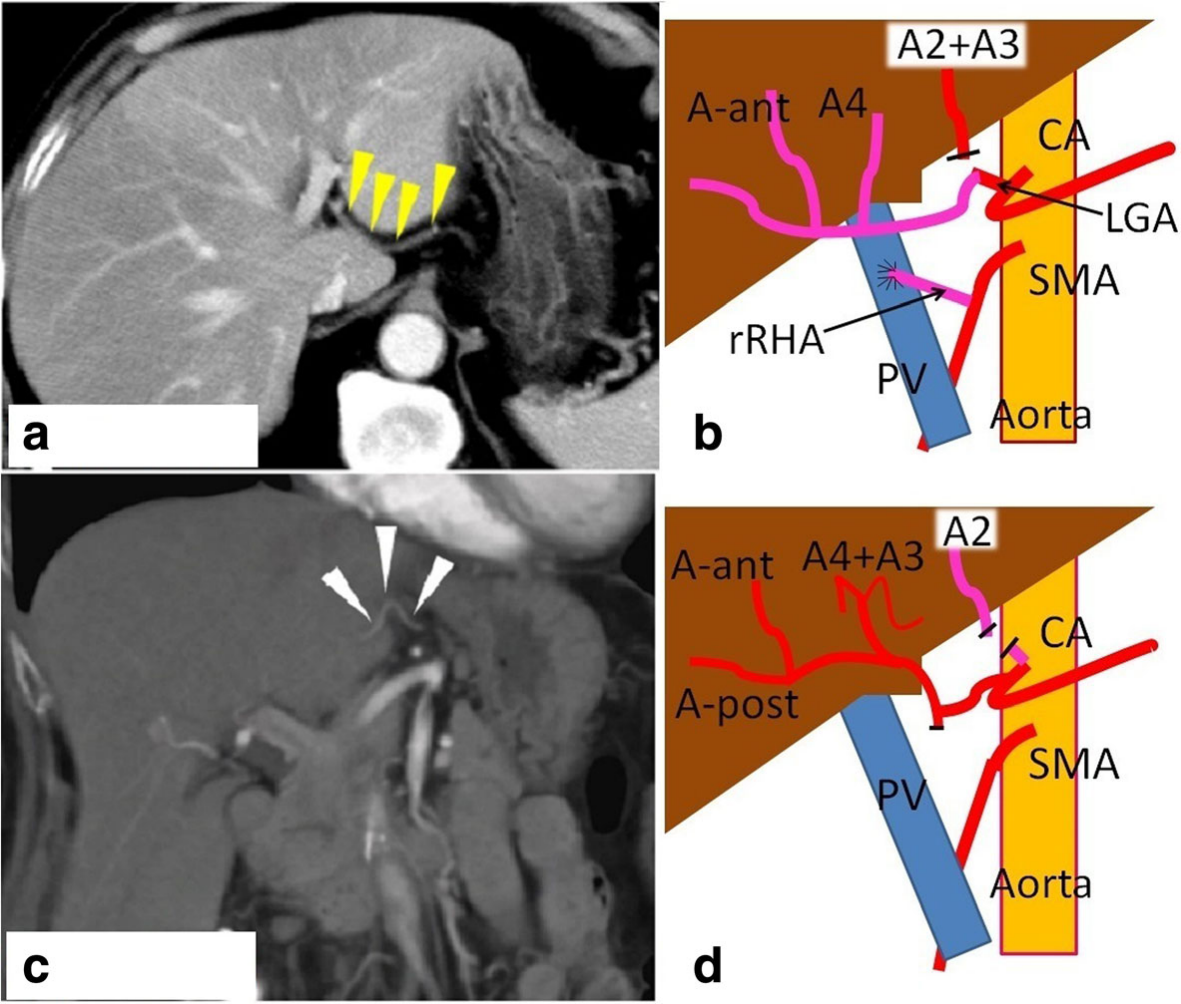
Preoperative CT and operative scheme for hepatic arteries following pancreaticoduodenectomy. **a** In case 1, the replaced left hepatic artery (rLHA) is segment 2/3 artery (A2 + A3) (*arrows*), which arises from the left gastric artery (LGA). **b** In case 1, Reconstructions of the hepatic arteries: the distal stump of the replaced right hepatic artery (rRHA) is anastomosed to the proximal stump of the LGA. The proximal stump of the rRHA is anastomosed with the left side of the portal vein (PV). The distal stump of the A2 + A3 is not reconstructed. **c** In case 2, the rLHA is segment 2 artery (A2) (*arrows*) only, which arises from the LGA. **d** In case 2, The distal stump of A2 is not reconstructed due to technical difficulties. *A-ant* anterior branch of the right hepatic artery, *A-post* posterior branch of the right hepatic artery, *A4* segment 4 artery, *A2 + A3* segment 2/3 artery, *CA* celiac axis, *LGA* left gastric artery, *rRHA* replaced right hepatic artery, *PV* portal vein, *SMA* superior mesenteric artery, *A4 + A3* segment 4/3 artery, *A2* segment 2 artery

A 66-year-old man with advanced gastric cancer that infiltrated the pancreatic head was referred to our department. Dynamic computed tomography (CT) revealed that the rLHA was a segment 2/3 artery (A2 + A3), which arose from a common channel from the LGA (Fig. 1a). The origin of A2 + A3 was encased by metastatic lymph nodes around the LGA. The rRHA originated from the SMA, and segment 4 artery (A4) was a branch of the rRHA. Moreover, the rRHA was surrounded and infiltrated by the tumor. The patient had no history of bile duct operations that could potentially cause biliary bacterial colonization. Preoperatively, he had no obstructive jaundice due to gastric cancer. No other medical history indicating a risk of ischemic liver injury was observed, such as coagulation abnormality, diabetes, and chronic liver disease. We performed PD with an en bloc resection of both the rLHA and rRHA. The distal stump of the rRHA was anastomosed with the proximal stump of the LGA. The distal stump of A2 + A3 was not reconstructed due to its small diameter (Fig. 1b). Alternatively, we created an arterioportal shunt by anastomosing the proximal stump of the rRHA on the left side of the portal vein. At the end of the operation, the surface color of the liver was normal and no demarcation line was observed. Intraoperative blood loss was 3416 mL, and the operation time was 562 min. Histopathology showed a pT4N2M0 adenocarcinoma (Union for International Cancer Control (UICC) classification); resection margins were free of carcinoma.

**Fig. 2:**
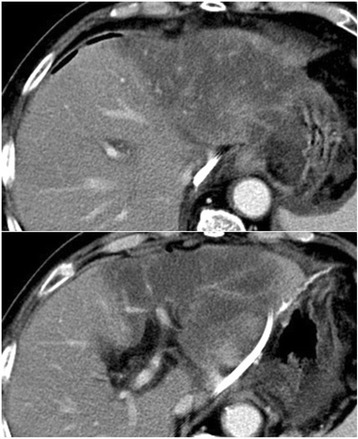
Case 1 CT findings 5 days after pancreaticoduodenectomy. Extensive necrosis is observed in the lateral segment of the liver

On postoperative day 1, serum aspartate transaminase (AST) and alanine transaminase (ALT) levels increased to 3450 and 3060 IU/L, respectively. On postoperative day 5, the patient went into septic shock and CT showed extensive necrosis in the lateral segment of the liver (Fig. 2). *Escherichia coli* was detected in blood culture. Due to poor physical condition of the patient, we could not perform liver resection. Postoperatively, maximum serum total bilirubin (T-Bil) and C-reactive protein levels were 9.5 and 30 mg/dL respectively. Biliary anastomotic leakage did not occur. The patient recovered with conservative treatment and was discharged 53 days after PD. Adjuvant chemotherapy was not administered. Since then, liver function had been normal during follow-up and biliary anastomotic stricture or ischemic cholangitis was not observed. CT at 1 year postoperatively revealed atrophy of the lateral segment and no cancer recurrence.

### Case 2

A 66-year-old woman with advanced gastric cancer that infiltrated the pancreatic head was referred to our department. CT revealed that the rLHA comprised segment 2 artery (A2) only, which arose from the LGA (Fig. 1c). Segment 3/4 artery was a common channel from the proper hepatic artery. The root of A2 was encased by metastatic lymph nodes. The patient had neither obstructive jaundice preoperatively nor a history posing the risk of postoperative liver necrosis. We performed PD with en bloc resection of the A2 without arterial reconstruction due to the small distal stump of A2 (Fig. 1d). At the end of the operation, the surface color of the liver was normal and there was no demarcation line observed. Intraoperative blood loss was 675 mL, and the operation time was 418 min. Histopathology showed a pT4N1M0 adenocarcinoma (UICC classification), and resection margins were tumor free.

**Fig. 3:**
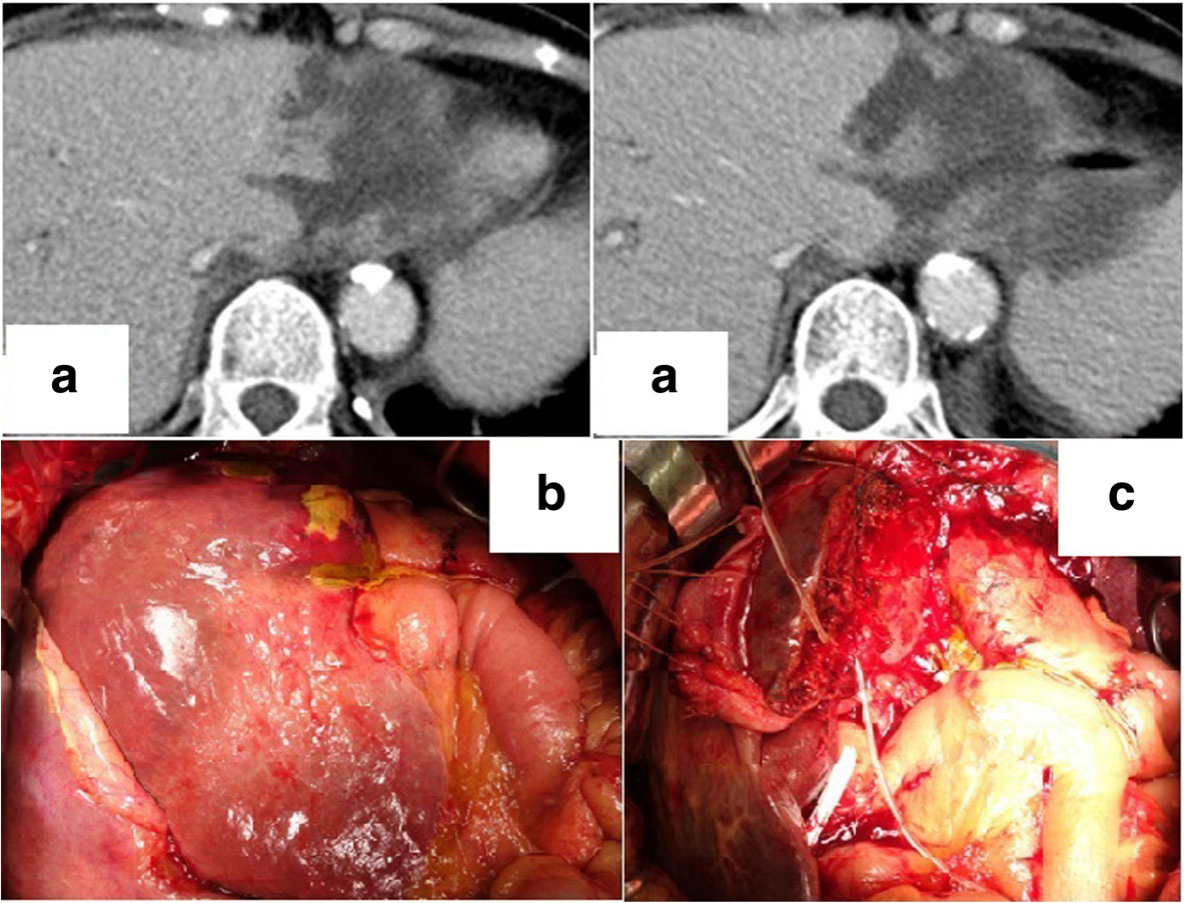
Case 2 CT findings 5 days after pancreaticoduodenectomy and relaparotomy findings. **a** Extensive necrosis is observed in the lateral segment of the liver. **b** The lateral segment becomes necrotic and is widely covered with yellow coat. **c** Lateral segmentectomy with external biliary drainage is performed

**Fig. 4:**
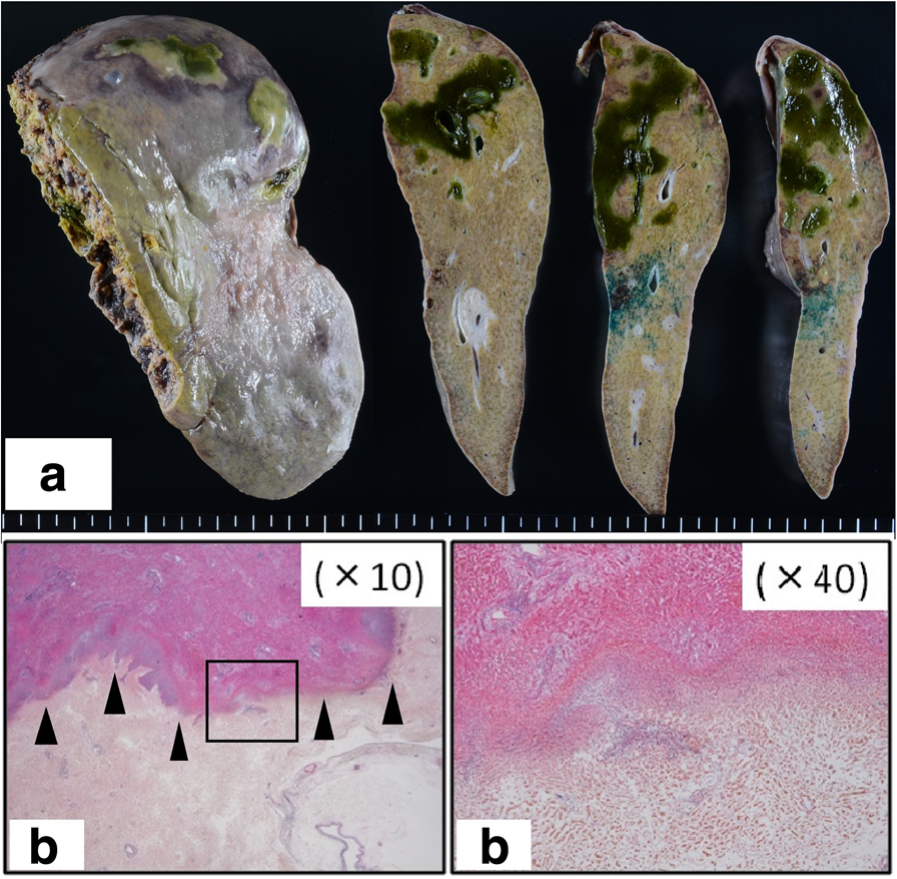
Pathological findings of the lateral segment in case 2. **a** There is liver necrosis in segment 2. **b** The border between normal and necrotic areas (*arrows*) is shown. The necrotic area is apparently circumscribed and confined to the segment 2 of the liver. There is abscess formation around the liver necrosis

On postoperative day 4, serum AST and ALT levels increased to 422 and 253 IU/L, respectively. On postoperative day 6, the patient went into septic shock and serum T-Bil and platelet counts were 6.6 mg/dL and 5.5 × 10^4^/μL, respectively. CT showed necrosis in the lateral segment of the liver (Fig. 3a). *Enterococcus faecium* was detected in blood culture. On postoperative day 7, we performed lateral segmentectomy of the liver with external biliary drainage (Fig. 3c). The lateral segment was widely covered with yellow coat and was necrotic (Fig. 3b). The biliary anastomosis did not leak. Pathologic examination of the resected lateral segment demonstrated necrosis with abscess formation (Fig. 4). The necrotic area was apparently circumscribed and confined to segment 2 area of the liver. Therefore, the liver necrosis was probably caused by A2 resection during PD. The patient subsequently recovered quickly and was discharged 38 days after PD. Adjuvant chemotherapy was not administered. Since then, she had an uneventful course and showed normal liver function in tests during follow-up. CT at 6 months after PD showed normal findings in the remnant liver and no cancer recurrence.

## Discussion and conclusions

During PD, particularly for gastric cancer, the rLHA arising from the LGA should be preserved as far as oncologically acceptable. Resection of the rLHA during PD can cause postoperative liver necrosis.

The liver can survive arterial ligation because of sufficient supply of oxygen and nutrients from the portal vein and several collateral arterial channel pathways [12]. A study on surgical anatomy based on dissection of 200 cadavers demonstrated 26 possible collateral arterial pathways to the liver [13]. Similarly, several radiologic studies showed many collateral arterial pathways in case of temporary interruption of blood supply from the hepatic artery [14–16]. Immediately after hepatic artery embolization (HAE), the following arteries that supply the liver have been radiologically identified: (1) inferior phrenic arteries through the right and left triangular and coronary ligaments, (2) branches of the superior and inferior epigastric arteries through the round and falciform ligaments, and (3) unnamed gastric collaterals in the lesser omentum and hepatoduodenal ligament [12, 13, 17].

For major hepato-biliary-pancreatic (HBP) surgeries including PD, extended hepatectomy, and hepato-pancreaticoduodenectomy (HPD), these abundant extrahepatic collateral arterial pathways are commonly divided by operative procedures, such as liver mobilization or dissection of the hepatoduodenal ligament [18]. Nevertheless, postoperative HAE for ruptured pseudoaneurysm had been considered safe and was widely performed even after a major HBP surgery [16, 18–20], probably because the remaining extrahepatic collateral pathways and the interlobar communicating artery (ICA) protect the liver from ischemic injuries [14, 16, 18–20].

The ICA runs through the Glissonean sheath around the bilateral hepatic duct confluence [14, 21, 22], which is known as the hilar plate [23], to perfuse liver areas without arterial supply [12, 14, 18, 21, 22]. Another study on seven cases of biliary tract carcinoma with unilateral hepatic artery involvement concluded that hepatic artery reconstruction was not always required as long as the hilar plate was preserved [21]. The ICA plays an important role in preventing ischemic injury from interruption of hepatic artery supply, particularly during a major HBP surgery [14, 21], and has been regarded an important communication into the liver [14, 15, 24, 25].

Conversely, an 18-case study [19] observed that 83% (15/18) developed ischemic liver injury after HAE for arterial hemorrhage following PD and HPD. However, most patients in that study had mild and transient ischemic liver damage and only one patient (7.7%) developed liver necrosis. The authors concluded that HAE following a major HBP surgery did not carry a great risk for serious ischemic liver injury.

Considering these evidences, arterial supply to the whole liver can be theoretically maintained after rLHA division during PD. Even if liver ischemic damage occurs, it would be mild and result in transient liver enzyme elevation. However, our two cases had serious postoperative liver necrosis. In both cases, the liver was not mobilized and the hilar plate was not dissected. The color of the liver surface was normal, and there was no demarcation line that appeared after rLHA division. In addition, b﻿lood flow from the residual hepatic arteries and portal vein was maintained perioperatively.

We assumed that the unexpected postoperative liver necrosis in this study was mainly caused by four possible mechanisms. First, the extent of lymph node dissection during PD for advanced gastric cancer tended to be larger than for other HBP diseases, such as those around the celiac axis and lesser omentum or those around the hepatoduodenal ligament; these may have reduced the extrahepatic collateral pathways to the liver. Some reports noted that extensive lymph node dissection around the liver could lead to liver necrosis after HAE following major HBP surgery [18, 26]. In that report, total remnant liver necrosis after HAE occurred in one patient (11%) who underwent whole liver mobilization during HPD [18]. In the field of liver transplantation, extrahepatic collateral pathways are absent immediately after operation and arterial supply depends on the reconstructed hepatic artery only [27, 28]. As a result, hepatic artery thrombosis can cause fatal liver necrosis [27, 28].

The second mechanism could be congenital underdevelopment of extrahepatic collateral pathways or the ICA. A report stated that the degree of collateral pathway development varied among individuals and that the course after HAE depended on the individual vascular anatomic characteristics [12]. A study on 16 adult cadaveric livers indicated that the ICA was not always existent and was not detected in 2/16 (13%) cases [25]. Indeed, a case report described liver necrosis with abscess formation in the lateral segment immediately after coil embolization of the rLHA [3]. The patient had not undergone any abdominal operation that potentially destroyed collateral arterial pathways around the liver. The author concluded that the liver necrosis was probably related to congenital underdevelopment of the ICA.

The third mechanism could be based on the fact that PD requires hepaticojejunostomy, in contrast to standard gastrectomy; this can cause upstream bacterial infection into the intrahepatic bile duct. To our knowledge, there had been no report on liver necrosis due to rLHA resection after standard gastrectomy for gastric cancer. A large-scale study on standard gastrectomy for 1340 consecutive patients with gastric cancer noted that all 116 cases that underwent rLHA resection did not have postoperative liver necrosis with abscess formation, although some of the patients had transient liver enzyme elevation [1]. Other studies on standard gastrectomy with rLHA resection reported the same outcomes in terms of ischemic liver injury [2, 4]. In our cases, biliary infection through hepaticojejunostomy may have led to liver necrosis with abscess formation in ischemic areas.

The fourth possible mechanism for the development of liver necrosis in our cases could be the reduction of total hepatic arterial flow due to rLHA resection with extended lymphadenectomy around the liver. Reportedly, sufficient hepatic arterial flow plays an important role in clearing bacterial translocation from the gut into the liver [29, 30]; that is, bacterial translocation under reduced hepatic arterial flow may have led to worse liver necrosis and abscess formation shortly after PD.

Although further work is required to clarify the potential risk factors of postoperative liver necrosis, we assumed that these four mechanisms mainly caused the unexpected postoperative liver necrosis in our patients, even though only the A2 + A3 or A2 was resected.

In usual clinical practice, it is impossible to identify whether these extrahepatic collateral pathways and the ICA are congenitally poor [19]. As a result, surgeons should always reconstruct the divided rLHA during PD [12]. However, microvascular reconstruction is technically demanding, particularly when the arterial stump is small, as in our cases. Judging from our clinical outcomes, we believe that surgeons should preserve the rLHA during PD as far as oncologically acceptable. In case the rLHA is resected during PD, surgeons should carefully observe the postoperative course of the patient while keeping in mind the possibility of urgent hepatectomy. If resection of the rLHA is inevitable due to tumor invasion, coil embolization of the rLHA before PD may be helpful in preventing postoperative ischemic complications, such as liver necrosis. In fact, a few case reports on requiring resection of rRHA from SMA stated that preoperative coil embolization of the rRHA prevented ischemic liver complications after PD [31–34].

In case 1, we created an arterioportal shunt as an alternative to microvascular reconstruction after A2 + A3 resection [35]. Although the effect remained unknown [35], the procedure may have contributed to the response to conservative management. In case 2, liver necrosis occurred even though only the A2 was resected. We presumed that the collateral pathways to the liver or the ICA were congenitally poor in this patient. In both cases, any demarcation line did not appear on the liver surface. We should have performed Doppler ultrasonography to confirm sufficient blood flow in the intrahepatic arteries.

To the best of our knowledge, this was the first report on liver necrosis shortly after PD due to the rLHA resection. This is potentially because the rLHA hardly interfered with operative procedure in PD for HBP disease [36], in contrast to that for gastric cancer. We consider it a pitfall that surgeons tend to underestimate the rLHA arising from the LGA [1, 2, 4], compared with the rRHA, particularly in gastric cancer surgery.

In conclusion, resection of the rLHA arising from the LGA during PD can cause postoperative liver necrosis, even if only the A2 + A3 or A2 was resected. We underscore that the rLHA should be preserved during PD as far as oncologically acceptable. In case rLHA resection is inevitable during PD due to tumor conditions, surgeons should carefully observe the patient in the postoperative course while keeping in mind the possibility of urgent hepatectomy.

## Abbreviations

A2: Segment 2 artery
A2 + A3: Segment 2/3 artery
A4: Segment 4 artery
ALT: Alanine transaminase
AST: Aspartate transaminase
CT: Computed tomography
HAE: Hepatic artery embolization
HBP: Hepato-biliary-pancreatic
HPD: Hepato-pancreaticoduodenectomy
ICA: Interlobar communicating artery
LGA: Left gastric artery
PD: Pancreaticoduodenectomy
rLHA: Replaced left hepatic artery
rRHA: Replaced right hepatic artery
SMA: Superior mesenteric artery
T-Bil: Total bilirubin
UICC: Union for International Cancer Control
